# *Toxoplasma gondii* Infection in Immunocompromised Patients: A Systematic Review and Meta-Analysis

**DOI:** 10.3389/fmicb.2017.00389

**Published:** 2017-03-09

**Authors:** Ze-Dong Wang, Huan-Huan Liu, Zhan-Xi Ma, Hong-Yu Ma, Zhong-Yu Li, Zhi-Bin Yang, Xing-Quan Zhu, Bin Xu, Feng Wei, Quan Liu

**Affiliations:** ^1^College of Life Science, Jilin Agricultural UniversityChangchun, China; ^2^State Key Laboratory of Veterinary Etiological Biology, Key Laboratory of Veterinary Parasitology of Gansu Province, Lanzhou Veterinary Research Institute, Chinese Academy of Agricultural SciencesLanzhou, China; ^3^Key Laboratory of Jilin Province for Zoonosis Prevention and Control, Military Veterinary Institute, Academy of Military Medical SciencesChangchun, China; ^4^Department of Emergency Medicine, Inner Mongolia General Forestry HospitalYakeshi, China; ^5^Medical Library of the Chinese people's Liberation ArmyBeijing, China; ^6^Center for Prevention and Control of Animal Diseases of Banan District in ChongqingChongqing, China; ^7^Key Laboratory of Zoonoses, Ministry of EducationChangchun, China

**Keywords:** *Toxoplasma gondii*, immunocompromised patients, HIV/AIDS patients, cancer patients, transplant recipients, prevalence, odds ratio

## Abstract

*Toxoplasma gondii* has been suggested as an important opportunistic pathogen in immunocompromised patients. We conducted a global meta-analysis to assess the prevalence and odds ratios (ORs) of *T. gondii* infection in immunocompromised individuals. Electronic databases were reviewed for *T. gondii* infection in HIV/AIDS patients, cancer patients, and transplant recipients, and meta-analyses were conducted to calculate overall estimated prevalence and ORs using random or fixed-effects models. Totally, 72 eligible studies were included. The estimated pooled prevalence of *T. gondii* infection in immunocompromised patients and the control was 35.9 and 24.7% (*p* < 0.001), with an OR of 2.24, i.e., 42.1 and 32.0% for HIV/AIDS patients and the control (*p* < 0.05), 26.0 and 12.1% for cancer patients and the control (*p* < 0.001), and 42.1 and 34.5% for transplant recipients and the control (*p* > 0.05), whose estimated pooled ORs were 1.92 (95% CI, 1.44–2.55), 2.89 (95% CI, 2.36–3.55), and 1.51 (95% CI, 1.16–1.95), respectively. This study is the first to demonstrate that the immunocompromised patients are associated with higher odds of *T. gondii* infection, and appropriate prevention and control measures are highly recommended for these susceptible populations.

## Introduction

The protozoan parasite *Toxoplasma gondii* can infect nearly all warm-blooded animals, including humans (Robert-Gangneux and Darde, 2012; Liu et al., 2015). Approximately 30% of the world's population is estimated to be infected with *T. gondii* (Montoya and Liesenfeld, 2004). Humans become primarily infected by ingesting raw or undercooked meat containing viable tissue cysts, or by ingesting water or food contaminated with oocysts from infected cat feces (Baldursson and Karanis, 2011; Meireles et al., 2015). In healthy humans, the infection with *T. gondii* is usually asymptomatic, but it can be fatal in the immunocompromised individuals, such as HIV/AIDS patients, cancer patients, and organ transplant recipients (Da Cunha et al., 1994; Pott and Castelo, 2013; Agrawal et al., 2014; Lu et al., 2015).

Toxoplasmosis of immunosuppressed individuals is most often the result of reactivation of latent infection, which presents neurological signs, including headache, disorientation, drowsiness, hemiparesis, reflex changes, and convulsions (Barratt et al., 2010; Robert-Gangneux and Darde, 2012). Acute acquired *T. gondii* infection in immunocompromised patients may also occur and involve multiple organs. Pneumonia, retinochoroiditis, and other disseminated systemic diseases, can also be seen, but are not as common as encephalitis in immunocompromised patients (Machala et al., 2015).

An increased frequency of *Toxoplasma* encephalitis has been reported in AIDS patients, especially those with significant immunosuppression when CD4 T lymphocyte cell counts is <200 cells/μL, and *T. gondii* infection is regarded as an important opportunistic pathogen that lead to the death of AIDS patients (Luft et al., 1993; Jones et al., 1996). The cancer can also reactivate latent *T. gondii* infection during antitumor treatment process (Frenkel et al., 1978). A variety of malignancies, including lymphoma, leukemia, and myeloma, can reactivate toxoplasmosis (Maciel et al., 2000; Kojima et al., 2010). Transplantation of an organ from seropositive donor can activate latent infection in a seronegative recipient receiving immunotherapy (Chehrazi-Raffle et al., 2015). Transplantation of an organ from seronegative donor can also initiate fatal infection by activation of the latent infection in a seropositive recipient receiving immunosuppressive therapy. It seems that danger of transplanting an infected organ into a seronegative recipient is greater than that of transplanting a non-infected organ into a seropositive recipient (Chehrazi-Raffle et al., 2015). Fatal toxoplasmosis has been reported in heart, liver and bone marrow, haematopoietic stem cell transplant recipients (Castagnini et al., 2007; Caner et al., 2008; Stajner et al., 2013; Gajurel et al., 2015).

Toxoplasmosis can be complicated and is considered a serious disease in immunocompromised patients, in which the reactivation of a latent infection can be fatal. The incidence of reactivated toxoplasmosis may rely on the prevalence and concentration of IgG antibodies (Robert-Gangneux and Darde, 2012). It is necessary to obtain information concerning the prevalence of *T. gondii* infection in different special populations worldwide. We conducted a global meta-analysis to assess the seroprevalence and odds ratios (ORs) of *T. gondii* infection in immunocompromised patients compared with those in control individuals.

## Materials and methods

### Search strategy and selection criteria

We reported this meta-analysis in accordance with the Preferred Reporting Items for Systematic Reviews and Meta-Analysis (PRISMA) statement (Moher et al., 2009). We searched PubMed, Embase, Google scholar, ScienceDirect, Chinese Web of Knowledge, Wanfang, and Chongqing VIP databases from inception to February 29, 2016, for all reports that possibly contained data for *T. gondii* prevalence in different immunocompromised populations. The databases were searched using the keywords “*Toxoplasma gondii*” and “toxoplasmosis” cross-referenced with “HIV,” “AIDS,” “acquired immune deficiency syndrome”, “cancer,” “tumor,” “malignancy,” “carcinoma,” “transplantation,” “organ grafting,” “immunodeficiency,” and “immune deficiency.” We included studies without language limitation.

We systematically searched the scientific literatures for case-control, cohort, and cross-sectional studies that reported *T. gondii* infection in immunocompromised individuals, stratified by one of the following criteria: population with HIV/AIDS or without HIV/AIDS; population with cancer or without cancer; transplant or non-transplant population. Studies were excluded if they were reviews, repeated studies, or animal studies. Studies were excluded if they provided the final result without raw data. Studies were excluded if the sample size from one of the two groups was <30.

All identified titles and abstracts were carefully examined by two independent reviewers (HHL and HYM). The full text of articles considered as potentially relevant based on title and abstract were independently examined by the same two reviewers. Any disagreements with the selected studies were resolved by discussion and the involvement of another two authors (ZDW and QL).

### Data extraction and quality assessment

The following information was extracted from each study: first author, publication year, country of the study, the number of patients and control, diagnostic methods, and demographic characteristics. Two reviewers (ZDW and YZL) independently extracted the data and reached a consensus after a discussion on the controversial literatures.

The quality of the included publications was assessed based on the criteria (Liu et al., 2009; Speich et al., 2016). These criteria were created based on the Grading of Recommendations Assessment, Development and Evaluation method (Atkins et al., 2004), and including the diagnostic approach of *T. gondii* infection and matching of case and control subjects (Table 1). A scoring approach was used for grading, and up to 11 points assigned to each study. Studies that were awarded 6–11 points were considered to be of high quality, 4–5 points were moderate quality, whereas lower scores indicated low quality.

**Table 1:** **Quality criteria for the included studies**.

**Quality parameter**	**Score**		
	**2**	**1**	**0**
Diagnostic approach	–	Approach clearly described	nd
	–	Repeatedly examined by a test or two different tests	nd
	–	Re-examined by a senior laboratory technician	nd
Study design	Cohort study	Case control study or cross sectional study	–
No. of case subjects	≥100	50–100	≤50
Source of population	Community-based or from two or more countries	≥2 hospitals	1 hospital
Matching of case and control subjects	Age and sex	Age or sex	nd

*nd, no data available*.

### Statistical analysis

We estimated prevalence of *T. gondii* infection by pooling of data from each study. Data were pooled with a DerSimonian-Laird random-effects model (DerSimonian and Laird, 1986; Borenstein et al., 2010), whose difference was compared using Wilcoxon two-sample test or *t*-test. The risk of *T. gondii* infection in patient and control groups was estimated by odds ratio (OR). It was considered statistically significant when *p* < 0.05. In the forest plots, *OR* > 1 showed a risk effect and *OR* < 1 showed a protective effect. Statistical heterogeneity of results was appraised using a *x*^2^-based *Q*-test and *I*^2^ statistic. The heterogeneity was considered not significant only when *p* > 0.1 and *I*^2^ < 50%. The fixed-effects model was used when literature heterogeneity not existed; otherwise, the random-effects model was employed. Sensitivity analysis was performed by modification of the inclusion criteria of this meta-analysis. The analysis was conducted using Stata software version 12.0 (Stata Corporation, College Station, TX, USA). The publication bias was considered significant when *p-*value of Begg's test and Egger's test was <0.05.

## Results

### Literature search

**Figure 1:**
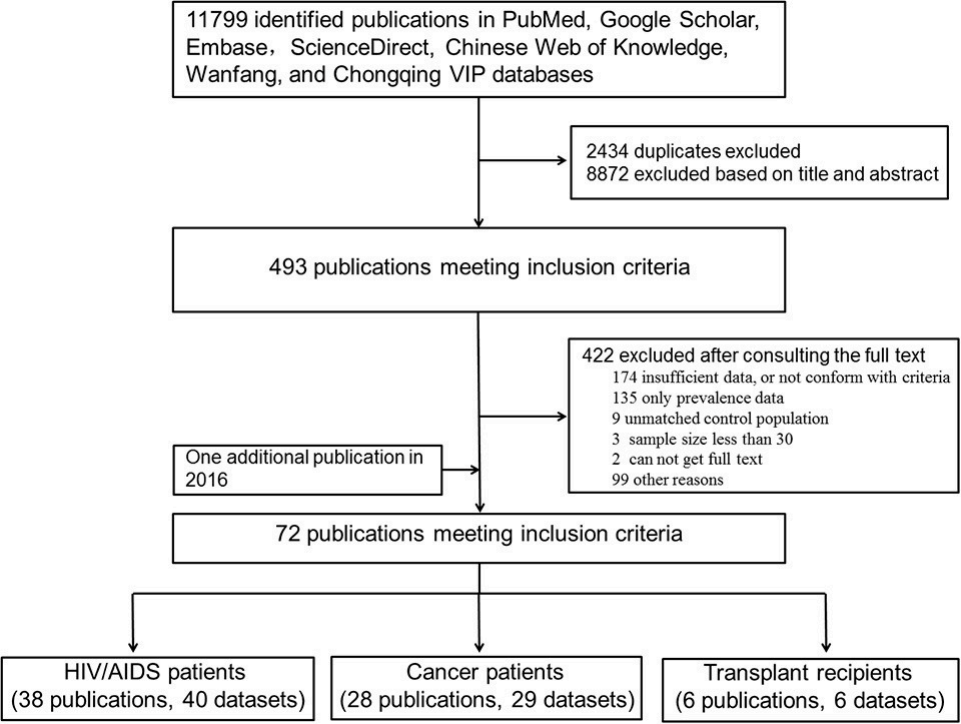
**Data search and selection**.

As shown in Figure 1, the literature search yielded 11,799 relevant studies, which included 2,434 duplicates. After a careful examination of each article's title and abstract, 493 were considered as having potential value, and the full texts were retrieved for detailed evaluation. A total of 422 potentially relevant articles were excluded from this meta-analysis after consulting the full text. Of these, 273 articles did not present sufficient data that required, or not conform with the included criteria; 135 had prevalence without raw data; 9 had unmatched control populations; the sample size in three articles was <30; there were two publications whose full texts were not retrieved. One additional publication regarding *T. gondii* infection in HIV patients was identified through the second search on Feb, 29, 2016. Finally, a total of 72 publications were included for our meta-analyses.

### Characteristics of included studies

**Figure 2:**
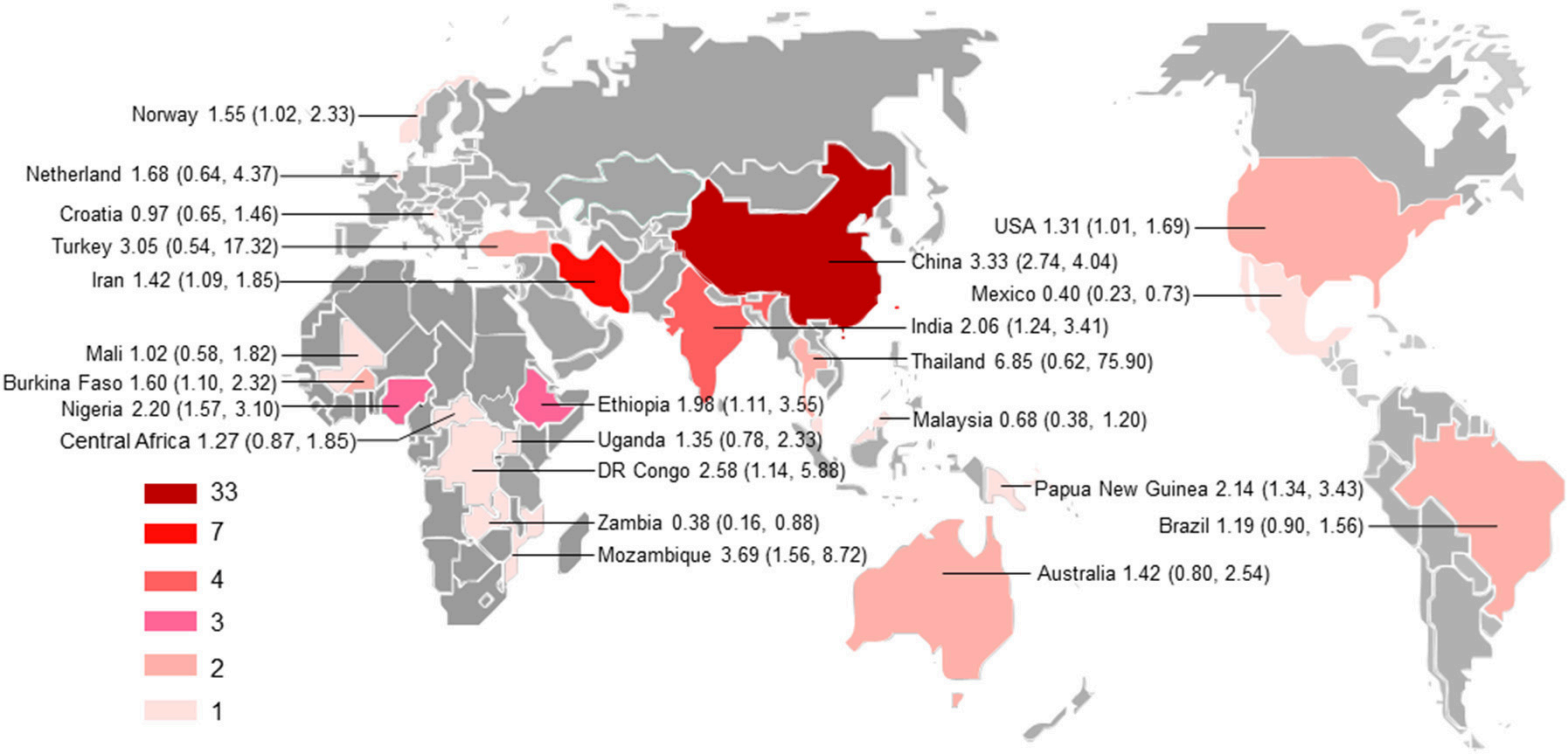
**Geographical distribution of the included studies**. The map was created using MapInfo Professional software version 9.5. Pooled odds ratio and 95% confidence interval are shown for each country.

Characteristics of the included publications are listed in Tables 2–**4**. In brief, 38 publications described *T. gondii* infection in HIV/AIDS patients, 28 articles investigated *T. gondii* infection in cancer patients, whereas 6 studies reported *T. gondii* infection in transplant patients. The identified studies were conducted worldwide (Figure 2). In terms of epidemiological design, 51 of the included publications were case-control studies, 17 were cross-sectional studies, and four were cohort studies. Thirty-nine papers were written in English, 29 were in Chinese, two in French (Maiga et al., 2001; Gamba et al., 2013), and one each in Spanish (Gongora-Biachi et al., 1998) and in Croatian (Dakovic-Rode et al., 2010). There were 6 papers whose raw data were extracted from the abstract (Ryan et al., 1993; Gongora-Biachi et al., 1998; Sukthana et al., 2000; Uneke et al., 2005; Akanmu et al., 2010; Manouchehri Naeini et al., 2015). The oldest study was conducted in 1987 (Quinn et al., 1987). Totally, 40 datasets investigated *T. gondii* infection in HIV/AIDS patients, and 29 datasets examined *T. gondii* infection in cancer patients, whereas only six datasets studied *T. gondii* infection in transplant recipients.

**Table 2:** **Characteristics of the included studies forT. gondii infection (IgG) in HIV/AIDS patients**.

**References**	**Study design**	**Country**	**Study population**	**Method**	**Score**
Quinn et al., 1987	C-C	DR Congo	NA	IFA	3
Quinn et al., 1987	C-C	USA	Homosexual men	IFA	4
Zumla et al., 1991	C-C	Uganda	NA	DT, LAT	6
Zumla et al., 1991	C-C	Zambia	NA	DT, LAT	6
Meisheri et al., 1997	C-C	India	NA	ELISA	2
Wongkamchai et al., 1995	C-C	Thailand	NA	ELISA	1
Gongora-Biachi et al., 1998	C-C	Mexico	NA	MEIA	4
Chaves-Borges et al., 1999	C-S	India	NA	ELISA	4
Sukthana et al., 2000	C-C	Thailand	NA	NA	3
Praharaj et al., 2001	C-C	India	NA	ELISA	7
Wanachiwanawin et al., 2001	C-C	Thailand	Pregnant women	ELISA	4
Maiga et al., 2001	C-C	Mali	NA	ELISA	5
Zhou and Huang, 2001	C-C	China	NA	MEIA	3
Falusi et al., 2002	C-S	USA	NA	DT	5
Nissapatorn et al., 2002	C-S	Malaysia	NA	ELISA	4
Uneke et al., 2005	C-C	Nigeria	NA	ELISA	4
Simpore et al., 2006	C-S	Burkina Faso	Pregnant women	ELISA	3
Jin et al., 2006	C-S	China	Drug user	ELISA	3
Shimelis et al., 2009	C-C	Ethiopia	NA	ELISA	5
Ouermi et al., 2009	C-C	Burkina Faso	Pregnant women	ELISA	5
Hua et al., 2009	C-C	China	NA	ELISA	3
Lago et al., 2009	C-S	Brazil	Pregnant women	ELFA	4
Akanmu et al., 2010	C-C	Nigeria	NA	ELISA	4
Li et al., 2010	C-S	China	Drug users	ELISA	3
Sitoe et al., 2010	C-C	Mozambique	Pregnant women	ELISA	4
Tian et al., 2010	C-C	China	NA	ELISA	6
Dakovic-Rode et al., 2010	C-C	Croatia	NA	ELISA	4
Daryani et al., 2011	C-S	Iran	NA	ELISA	4
Fernandes et al., 2012	C-C	Brazil	Pregnant women	ELFA	3
Song, 2012	C-S	China	NA	ELISA	5
John et al., 2012	C-C	Papua New Guinea	NA	ELISA	7
Alavi et al., 2013	C-C	Iran	Drug user	ELISA	2
Gamba et al., 2013	C-C	Central Africa	Pregnant women	ELISA	5
You, 2013	C-C	China	NA	ELISA	7
Ogoina et al., 2013	C-S	Nigeria	NA	ELISA	3
Walle et al., 2013	C-S	Ethiopia	NA	ELISA	5
Endris et al., 2014	C-S	Ethiopia	NA	ELISA	2
Pang et al., 2015	C-S	China	NA	ELISA	3
Uppal et al., 2015	C-S	India	NA	ELISA	4
Shen et al., 2016	C-C	China	NA	ELISA	3

*HIV, human immunodeficiency virus; AIDS, acquired immune deficiency syndrome; C-C, case control study; C-S, cross-sectional study; NA, not applicable because the reference does not provide this parameter; IFA, indirect fluorescent antibody test; MEIA, microparticle enzyme immunoassay; DT, dye test; LAT, latex agglutination test; ELISA, enzyme-linked immunosbsorbent assay*.

According to our criteria, eight publications were of high quality (>6 points), 43 publications had quality scores of 4–6 points indicating moderate quality, whereas the remaining 21 publications were of low quality (<4 points).

### Pooled prevalence of *T. gondii* infection (IgG) in immunocomprised patients

The estimated pooled prevalence of *T. gondii* infection in the HIV/AIDS patients and control population was 42.1% (95% CI, 34.0–50.2%) and 32.0% (95% CI, 24.0–40.1%), respectively (*p* < 0.05); the prevalence in cancer patients and control was 26.0% (95% CI, 20.5–31.5%) and 12.1% (95% CI, 9.5–14.8%), respectively (*p* < 0.001); and the prevalence in transplant recipients and its control was 42.1% (95% CI, 27.1–57.2%) and 34.5% (95% CI, 17.1–51.9%), respectively (*p* = 0.59). The results are shown in Supplementary Figures 1–6.

### Association of immunocomprised patients with *T. gondii* infection

**Figure 3:**
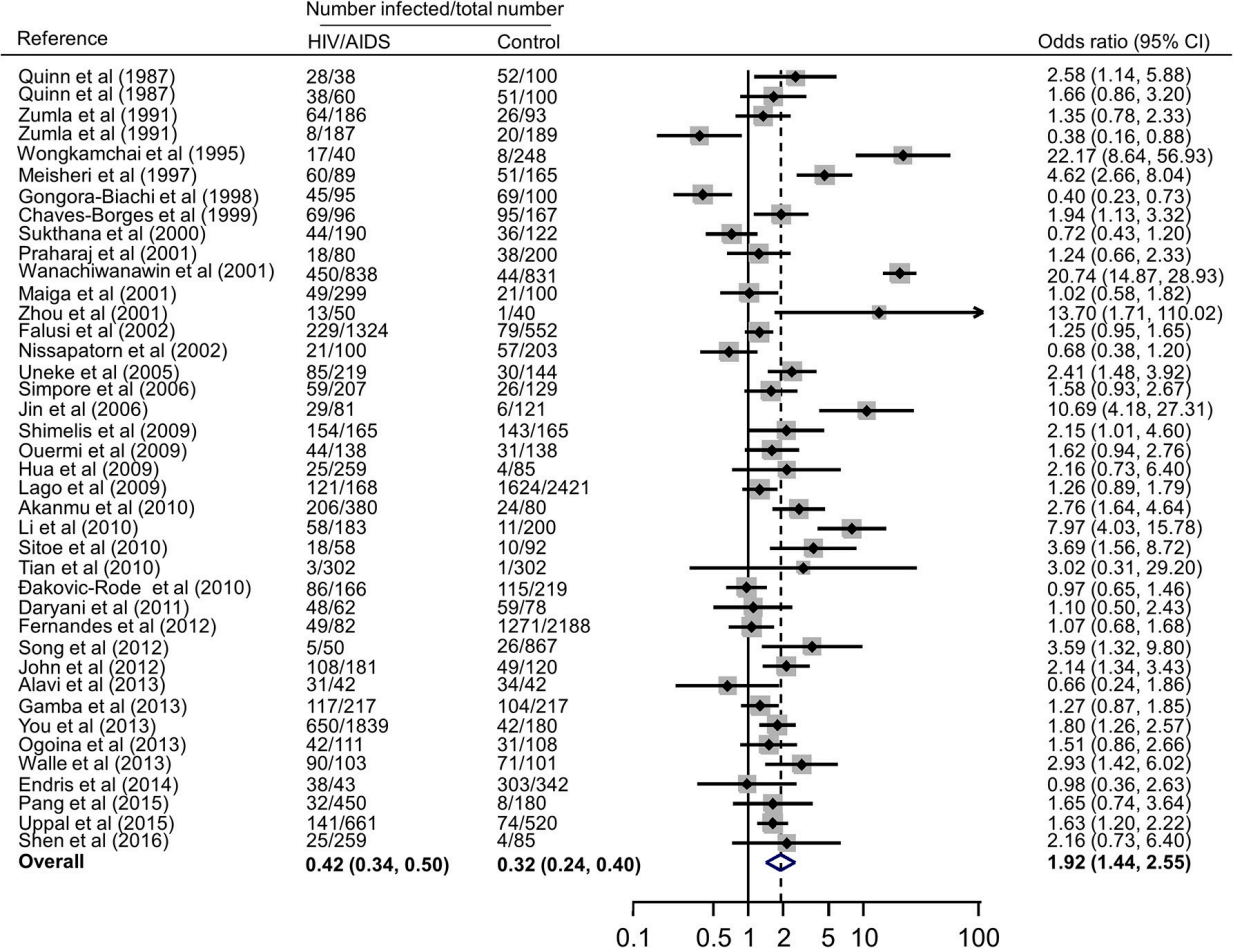
**Meta-analysis of the association of HIV/AIDS patients and ***T. gondii*** infection (IgG) with random-effects analysis**. CI, confidence interval; OR, odds ratio.

**Figure 4:**
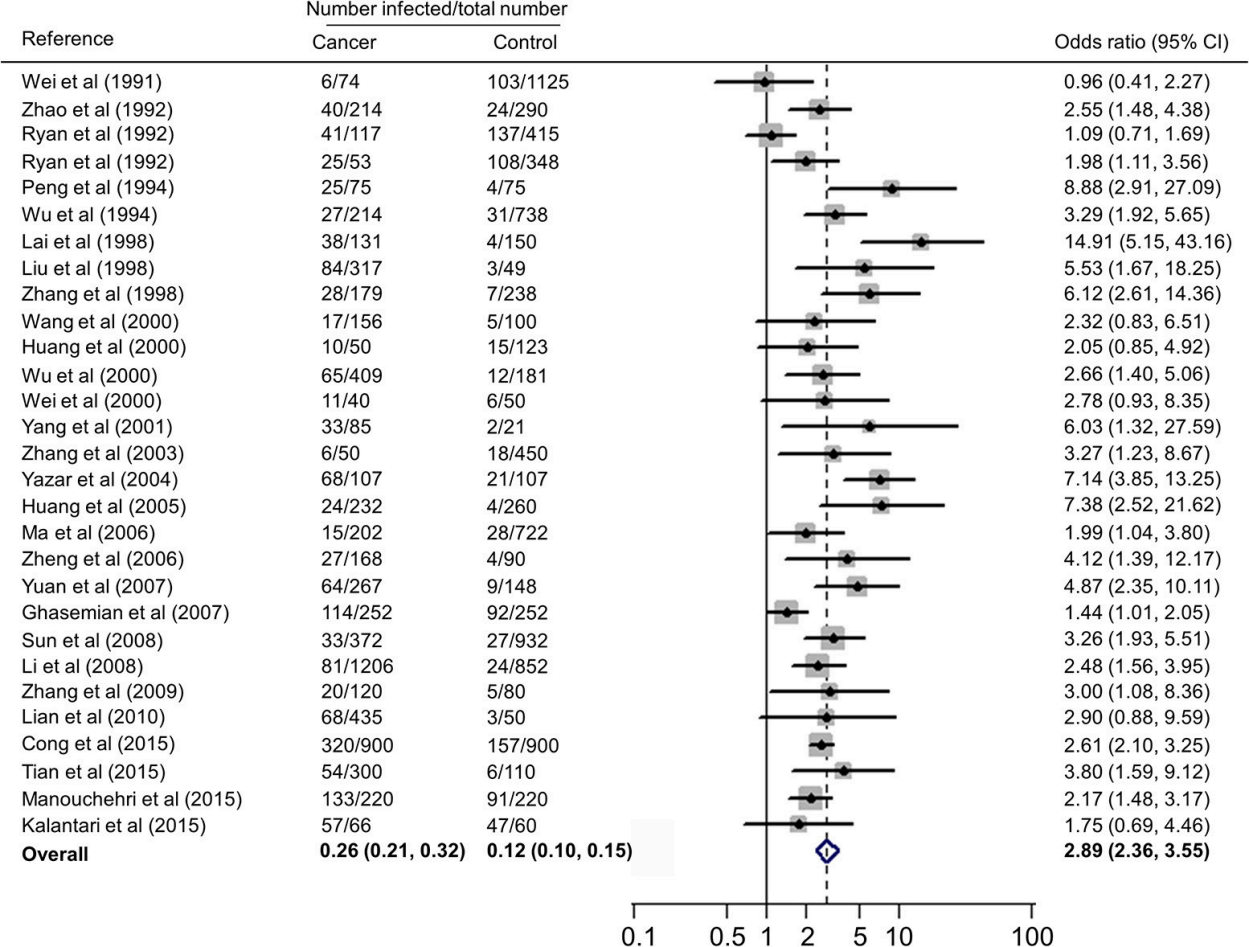
**Meta-analysis of the association of cancer patients and ***T. gondii*** infection (IgG) with random-effects analysis**. CI, confidence interval; OR, odds ratio.

**Figure 5:**
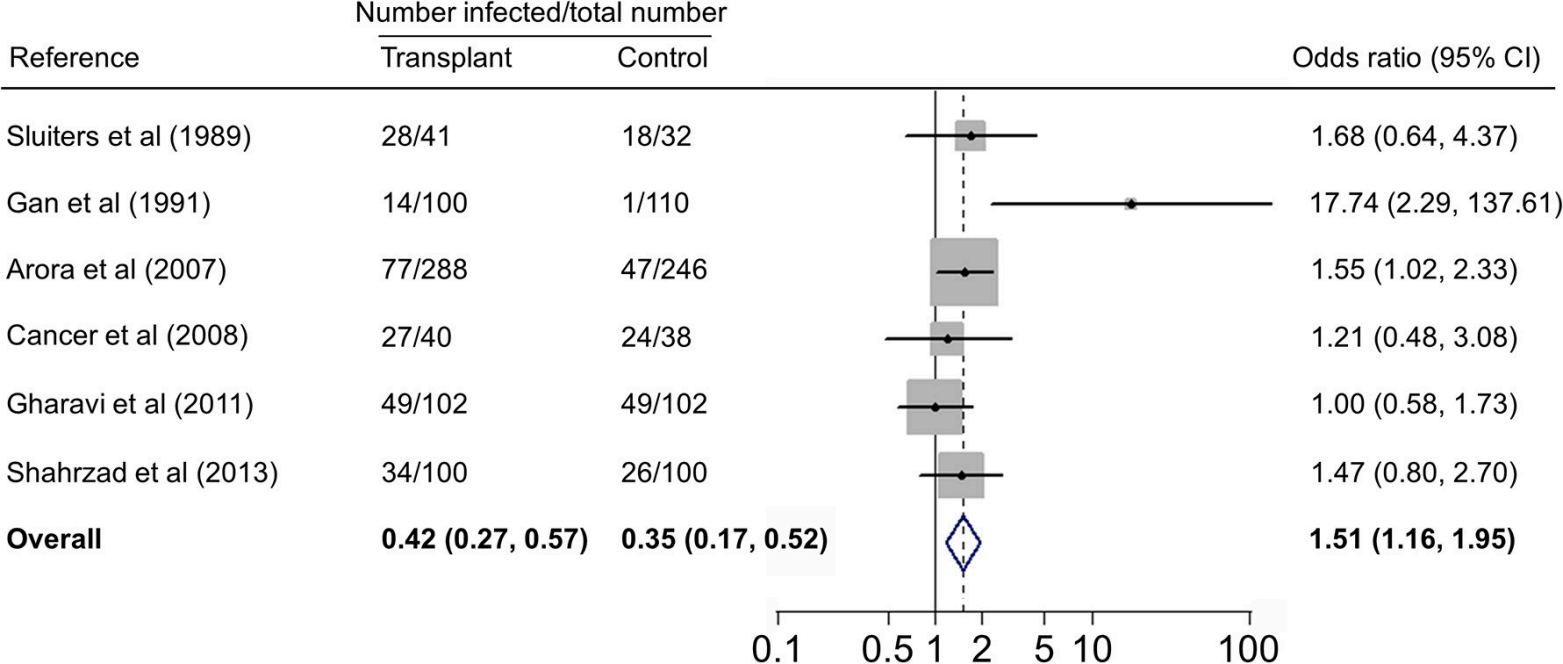
**Meta-analysis of the association of transplant recipients and ***T. gondii*** infection (IgG) with fixed-effects analysis**. CI, confidence interval; OR, odds ratio.

Forest plots on the association of immunosuppressed populations with *T. gondii* infection are presented in Figures 3–5. The estimated pooled random effects ORs of HIV/AIDS, cancer, and transplant patients compared with their controls were 1.92 (95% CI, 1.44–2.55), 2.89 (95% CI, 2.36–3.55), and 1.51 (95% CI, 1.16–1.95) for infection with *T. gondii*. However, the heterogeneity analysis showed that there was a relatively high-level heterogeneity in our meta-analysis of HIV/AID patients (*Q* = 401.6, *I*^2^ = 90.3%, *p* = 0.000) and cancer individuals (*Q* = 76.4, *I*^2^ = 63.4%, *p* = 0.000), and no heterogeneity was found in transplant recipients (*Q* = 8.0, *I*^2^ = 37.3%, *p* = 0.157).

We also analyzed all the data, showing that the estimated pooled prevalence of *T. gondii* infection in immunocompromised patients and the controls was 35.9% (95% CI, 31.0–40.8%) and 24.7% (95% CI, 20.5–28.8%, *p* < 0.001), with an OR of 2.24 (95% CI, 1.87–2.69).

### Subgroup analysis

All subgroup analysis, including those of geographical distribution, country income, published years, sample size, detection methods, study design, and population, did not show any significant differences between the respective groups, as indicated by overlapping 95% CIs, with the exception of subgroups based on geographical distribution and income level (Supplementary Table 1). For example, the odd of *T. gondii* infection in HIV/AIDS patients in Asia (*OR* = 2.77, 95% CI, 1.58–4.87) was significantly higher as comparison with that in Latin America (*OR* = 1.19, 95% CI, 0.90–1.56) and in Europe (*OR* = 0.97, 95% CI, 0.65–1.46); the odd of *T. gondii* infection in cancer patients in Asia (*OR* = 3.07, 95% CI, 2.51–3.76) was significantly higher, compared with that in Oceania (*OR* = 1.42, 95% CI, 0.80–2.54). Additionally, higher odds of *T. gondii* infection in both HIV/AIDS and cancer patients were found in middle-income and low-income countries, compared with that of high-income countries.

### Publication bias and sensitivity analysis

Begg and Egger tests were used to evaluate the publication bias. No significant bias was revealed in HIV/AIDS- or transplant-related publications, but significant bias was observed in cancer-associated publications (*p* < 0.05, Supplementary Table 1, Supplementary Figure 7).

A sensitivity analysis was conducted by excluding one single study each time to find out whether modification of the inclusion criteria of this meta-analysis had an effect on the final results. All the results were not materially altered (data not shown).

### Pooled prevalence of *T. gondii* infection (IgM) in immunocomprised patients

Our meta-analysis focused on *T. gondii* IgG antibodies, which are a marker of lifetime exposure to toxoplasmosis, whereas IgM antibodies are a marker of acute or recent infection, or also potentially persistent infection or reinfection with a different genotype (Sharma et al., 1983; Dzitko et al., 2006). During extraction of data in this study, the IgM antibodies against *T. gondii* in immunocompromised patients were also collected (Supplementary Tables 2–4). Due to insufficient data on HIV/AIDS and transplant patients, we only analyzed *T. gondii* IgM in cancer patients and the control, showing a prevalence of 11.4% (95% CI, 8.1–14.7%) in cancer patients and 2.7% (95% CI, 1.5–4.0%, *p* < 0.01) in its control group and OR of 2.65 (95% CI, 2.04–3.45, Supplementary Figures 8–10). The results also confirmed that the immunocompromised patients were associated with significantly higher odds of recently acquired *T. gondii* infection.

## Discussion

*T. gondii* has been suggested as an important opportunistic pathogen in immunocompromised patients (Walker and Zunt, 2005). The infection in healthy (immunocompetent) people is usually self-limited and asymptomatic, resulting in chronic infection of tissue cysts that can lie dormant, probably for the entire lifetime of the hosts. However, immunocompromised individuals, such as HIV/ADIS patients, cancer patients with chemotherapy, and transplant recipients, are at risk of developing *Toxoplasma* encephalitis, myocarditis, or pneumonitis, due to reactivation of the chronic infection. For example, approximately 30–40% of HIV co-infected immunocompromised individuals with *T. gondii* develop encephalitis (Walker and Zunt, 2005).

The associations of HIV and seroprevalence of *T. gondii* infection are varied in the world (Grant et al., 1990). Some reports showed higher prevalence of *T. gondii* infection in HIV-infected patients compared to non-infected individuals, whereas others did not find any differences between the two groups (Sukthana et al., 2000; Galvan-Ramirez Mde et al., 2012). This global systematic review and meta-analyses were conducted to quantify the prevalence and ORs of *T. gondii* infection in immunocompromised individuals compared with those in control individuals.

Subgroup analyses comparing published year, sample size, detection method, study design, country income, and population revealed non-significant differences, but high odds were found for *T. gondii* infection in HIV/AIDS patients in Asia and Africa as comparison with that of America and Europe, and in cancer patients in Asia compared to that in Oceania (Supplementary Table 1). However, only one or two studies examined the association of immunocompromised patients with *T. gondii* infection in these regions. Thus, no meta-analysis could be done and no firm conclusion should be drawn. Most studies were conducted in the countries of Asia. Our analyses further demonstrated that the studies are geographically clustered, with few studies in Latin America, Europe, and Oceania (Figure 2).

The presence of heterogeneity was observed in HIV/AIDS and cancer patients, but subgroup analyses did not explain the specific causes of heterogeneity, which may come from various sources, including geographical distribution, published years, sample size, detection methods, study design, or populations. Without meta-regression or additional subgroup analysis that requires a large number of studies, it is difficult to investigate the causes of heterogeneity. The presence of heterogeneity shows that pooled results are averaging multiple related, but different effects (Strunz et al., 2014).

In fact, higher prevalence of *T. gondii* infection in HIV/AIDS patients has been reported in many countries, such as Nigeria, Mali, Ethopia, India, China, and Thailand (Maiga et al., 2001; Wanachiwanawin et al., 2001; Akanmu et al., 2010; Daryani et al., 2011; Uppal et al., 2015; Shen et al., 2016). The present study provided robust evidence that support the conclusion, and demonstrated that HIV/AIDS patients are associated risk factors (*OR* = 1.92, 95% CI 1.44–2.55) for *T. gondii* infection. The data were derived from 38 publications from 20 countries, which included 10,028 HIV/AIDS patients and 12,334 control people (Table 2).

A recent study reported *T. gondii* infection in Chinese cancer population, with a prevalence of 20.6% in cancer patients and 6.3% in the control (*OR* = 3.9) (Jiang et al., 2015). Our meta-analysis also included the data of other countries, such as Australia, Iran, and Turkey (Table 3), which involved 7,011 cancer patients and 9,254 control people, therefore, the results would be more reliable. However, of the included 28 publications, 21 were written in Chinese, resulting in significant publication bias.

**Table 3:** **Characteristics of the included studies for cancer patients**.

**References**	**Study design**	**Country**	**Cancer**	**Control**	**Method**	**Score**
Wei et al., 1991	C-C	China	Mixed	NP	IHA, ELISA	4
Zhao et al., 1992	C-C	China	Mixed	NP	ELISA	2
Ryan et al., 1993	C-C	Australia	Glioma	NP	ELISA	4
Ryan et al., 1993	C-C	Australia	Meningioma	NP	ELISA	4
Peng et al., 1994	C-C	China	Mixed	NP	IHA	4
Wu et al., 1994	C-C	China	Mixed	NP	IHA	5
Lai et al., 1998	C-C	China	Mixed	NP	ELISA	5
Liu and Li, 1998	C-C	China	Mixed	NP	IHA	3
Zhang et al., 1998	C-S	China	Mixed	NP	IHA	5
Wang et al., 2000	C-C	China	Mixed	NP	ELISA	6
Huang et al., 2000	C-C	China	Cervical cancer	Other diseases	ELISA	3
Wu et al., 2000	C-C	China	Mixed	NP	IHA, ELISA	5
Wei and Zhu, 2000	C-C	China	Mixed	NP	ELISA	3
Yang et al., 2001	C-C	China	Mixed	NP	ELISA	4
Zhang et al., 2003	C-C	China	Mixed	NP	ELISA	3
Yazar et al., 2004	C-C	Turkey	Mixed	NP	ELISA	7
Huang et al., 2005	C-C	China	Mixed	NP	IHA	5
Ma et al., 2006	C-C	China	Mixed	NP	ELISA	3
Zheng et al., 2006	C-C	China	Lung cancer	NP	ELISA	5
Yuan et al., 2007	C-C	China	Mixed	NP	ELISA	4
Ghasemian et al., 2007	C-S	Iran	Mixed	NP	ELISA	7
Sun et al., 2008	C-C	China	Mixed	NP	ELISA	4
Li et al., 2008	C-C	China	Mixed	NP	ICT	4
Zhang et al., 2009	C-C	China	Mixed	NP	ELISA	3
Lian et al., 2010	C-C	China	Mixed	NP	ELISA	4
Cong et al., 2015	C-C	China	Mixed	NP	ELISA	8
Tian et al., 2015	C-C	China	Leukemia and Lymphoma	NP	ELISA	3
Manouchehri Naeini et al., 2015	C-C	Iran	Mixed	NP	ELISA	7
Kalantari et al., 2015	C-S	Iran	Breast cancer	Healthy women	ELISA	6

*C-C, case-control study; C-S, cross-sectional study; IHA, indirect hemagglutination; ELISA, enzyme-linked immunosbsorbent assay; ICT, immunochromatographic test; NP, normal population*.

There are many case reports of toxoplasmosis in transplant recipients, including haematopoietic cell (Barcan et al., 2002), heart (Gajurel et al., 2015), liver (Hamza et al., 2015), and kidney (Petty et al., 2015) transplant patients. A previous study reported a higher prevalence of *T. gondii* infection in renal transplant recipients (Soltani et al., 2013). In the present study, we analyzed *T. gondii* infection in 671 transplant patients (heart, kidney, and liver) and 628 control people from five countries (Table 4), revealing no significant difference of *T. gondii* infection between the two groups, but showing that transplant population is an risk factor (*OR* = 1.51, 95% CI, 1.16–1.95) for *T. gondii* infection. Interestingly, it was found that 14.3% of renal transplant recipients were detected positive for *T. gondii* infection in the first year of transplantation, and the prevalence increased to 85.7% in 1 year post-transplantation (Aufy et al., 2009).

**Table 4:** **Characteristics of the included studies for transplant recipients**.

**References**	**Study design**	**Country**	**Control population**	**Transplanted organ**	**Method**	**Score**
Sluiters et al., 1989	C	Netherland	Donor	Heart	ELISA	5
Gan et al., 1991	C-C	China	Self-control	Kidney	IHA	4
Arora et al., 2007	C	Norway	Donor	Heart	ELISA	5
Caner et al., 2008	C	Turkey	Donor	Liver	DT	5
Gharavi et al., 2011	C	Iran	Self-control	Kidney	ELFA, ELISA	9
Gharavi et al., 2011	C	Iran	Self-control	Kidney	ELISA	9
Soltani et al., 2013	C-S	Iran	Healthy subjects	Kidney	ELISA	4

*C, cohort study; C-C, case-control study; C-S, cross-sectional study; IHA, indirect hemagglutination; DT, dye test; LAT, latex agglutination test; ELFA, enzyme-linked flourescence assay; ELISA, enzyme-linked immunosorbent assay*.

There are several limitations in this meta-analysis, which may affect the results. First, a number of potentially relevant studies were identified through our systematic review, but not all the underlying data were available. Therefore, though most of these studies might not have relevant data, there is a certain risk to miss some eligible data.

Second, based on our scoring system, most studies were of moderate or even relatively low quality. This finding is mainly due to the epidemiological design of the studies; most were cross-sectional in nature. The differences between the study groups also included ages, lifestyles, and geographical conditions, which all contribute to the difference of *T. gondii* infection between the patient and control groups (Minbaeva et al., 2013; Walle et al., 2013; Wang et al., 2015). The cluster randomized controlled trial would provide high-quality data, but their implementation is more difficult. An alternative way to generate high-quality data would be using a time-series approach as a study design (Speich et al., 2016).

Third, diagnosis of *T. gondii* infection in immunocompromised patients is difficult. Though detection of the parasite by microscopy and bioassays is considered as the gold standard for diagnosis of toxoplasmosis, its clinical diagnosis more likely relies on serological methods (Liu et al., 2015). However, the serological methods may be unreliable in the immunocompromised individuals, whose immune system has been impaired, and cannot produce enough antibodies (Lewis et al., 2015). In the identified studies, all the detection methods were serological, including indirect hemagglutination (IHA), dye test (DT), immunochromatographic test (ICT), and enzyme-linked immunosorbent assay (ELISA) (Tables 2–4). Thus, by the reason of lack of specific antibody, the detected results would be lower than the actual prevalence in immunocompromised patients, including HIV/AIDS patients, cancer patients, and transplant recipients (Saadatnia and Golkar, 2012).

Fourth, insufficient data about further relevant factors on *T. gondii* infection (e.g., age, cancer type, transplanted organ) were available for subgroup analysis.

In summary, our global meta-analysis shows a higher prevalence of *T. gondii* infection in immunocompromised patients, and demonstrates that the immunocompromised individuals, including HIV/AIDS patients, cancer patients, and transplant recipients, were associated with higher odds of *T. gondii* infection. Therefore, a routine serological screening test for *T. gondii* infection is suggested to be conducted in immunocompromised patients in endemic area, or patients with no proper chemoprophylaxis and/or HAART. Patients with a positive result are at risk of reactivation of the infection, while patients with a negative result should be informed to prevent primary infection. Health education, particularly toward avoiding eating raw and undercooked meat, and avoiding contact with cats' feces should also be considered.

## Author contributions

QL was responsible for the idea and concept of the paper. ZW and QL analyzed the results. HL, ZM, HM, ZL, FW, ZY, and BX collected and analyzed the data. QL and XZ wrote the manuscript. All authors contributed to the manuscript editing and approved the final manuscript.

## Funding

This work was supported by the National Natural Science Foundation of China (31672542, 31472183, 31372430 and 31230073) and the Special Fund for Agro-scientific Research in the Public Interest in China (201303042).

### Conflict of interest statement

The authors declare that the research was conducted in the absence of any commercial or financial relationships that could be construed as a potential conflict of interest.

## Supplementary material

Supplementary Figure 1 **Forest plot of estimated pooled prevalence (IgG) of ***T. gondii*** infection in HIV/AIDS patients**.

Supplementary Figure 2 **Forest plot of estimated pooled prevalence (IgG) of ***T. gondii*** infection in HIV/AIDS-negative population**.

Supplementary Figure 3 **Forest plot of estimated pooled prevalence (IgG) of ***T. gondii*** infection in cancer patients**.

Supplementary Figure 4 **Forest plot of estimated pooled prevalence (IgG) of ***T. gondii*** infection in cancer-negative patients**.

Supplementary Figure 5 **Forest plot of estimated pooled prevalence (IgG) of ***T. gondii*** infection in transplant patients**.

Supplementary Figure 6 **Forest plot of estimated pooled prevalence (IgG) of ***T. gondii*** infection in non-transplant population**.

Supplementary Figure 7 **Funnel plots to assess publication bias in the meta-analysis. (A)** Funnel plot for experimental studies. **(B)** Funnel plot for observational studies.

Supplementary Figure 8 **Meta-analysis of the association of cancer patients and ***T. gondii*** infection (IgM)**.

Supplementary Figure 9 **Forest plot of estimated pooled prevalence (IgM) of ***T. gondii*** infection in cancer patients**.

Supplementary Figure 10 **Forest plot of estimated pooled prevalence (IgM) of ***T. gondii*** infection in cancer-negative population**.

Supplementary Table 1 **Results of the subgroup analyses examining the association of immunosuppressed individuals with ***T. gondii*** infection^*****^**.

Supplementary Table 2 **Characteristics of the included studies for ***T. gondii*** infection (IgM) in HIV/AIDS patients**.

Supplementary Table 3 **Characteristics of the included studies for ***T. gondii*** infection (IgM) in cancer patients**.

Supplementary Table 4 **Characteristics of the included studies for ***T. gondii*** infection (IgM) in transplant patients**.
