# Supplementary figures and images for: Toxoplasma gondii Infection in Immunocompromised Patients: A Systematic Review and Meta-Analysis
Source: Front Microbiol. 2017 Mar 9;8:389. doi: 10.3389/fmicb.2017.00389 (PMC5343064; doi:10.3389/fmicb.2017.00389)

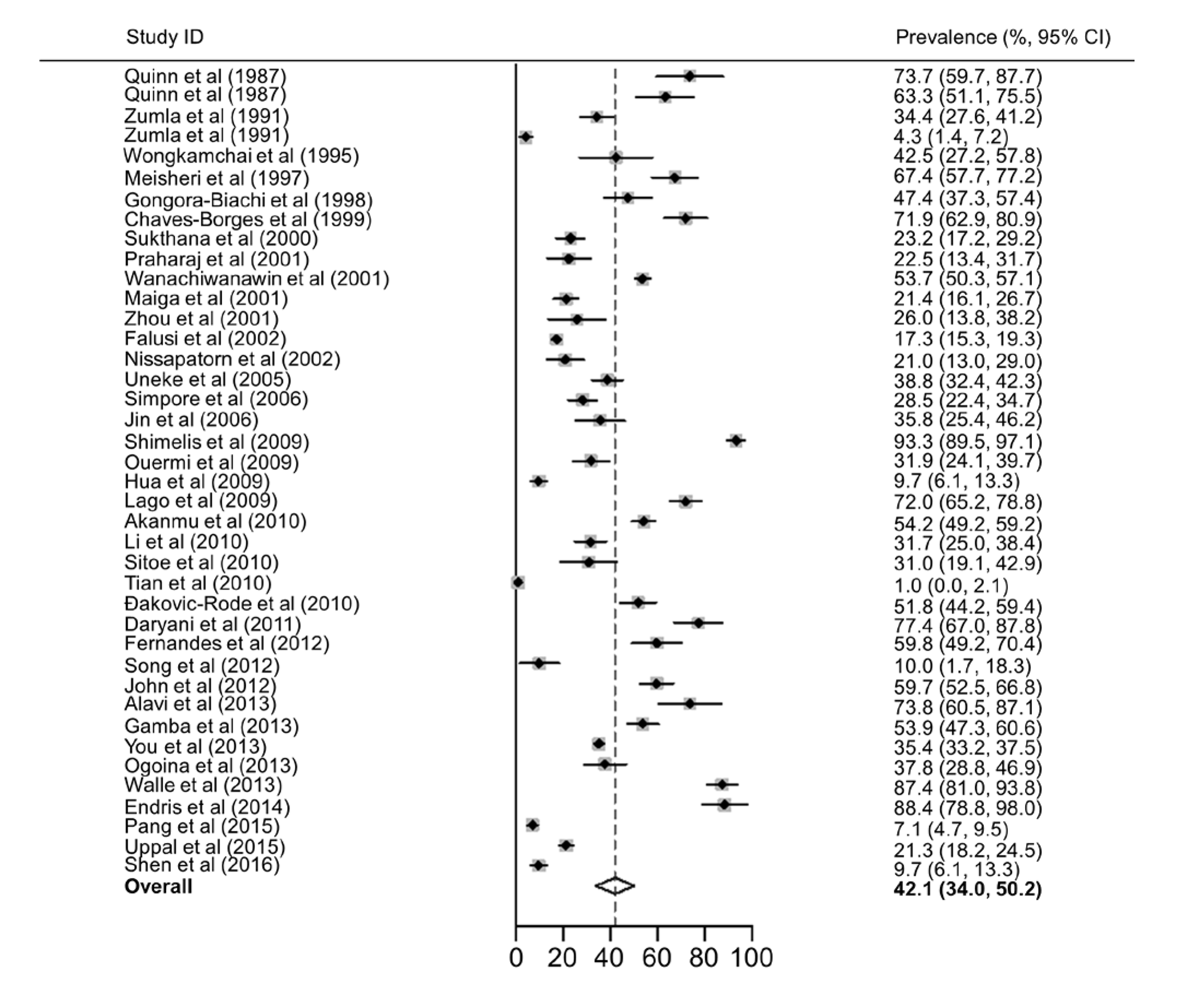

Supplement: Supplementary Figure 1 — Forest plot of estimated pooled prevalence (IgG) of T. gondii infection in HIV/AIDS patients. [file Image1.TIF]

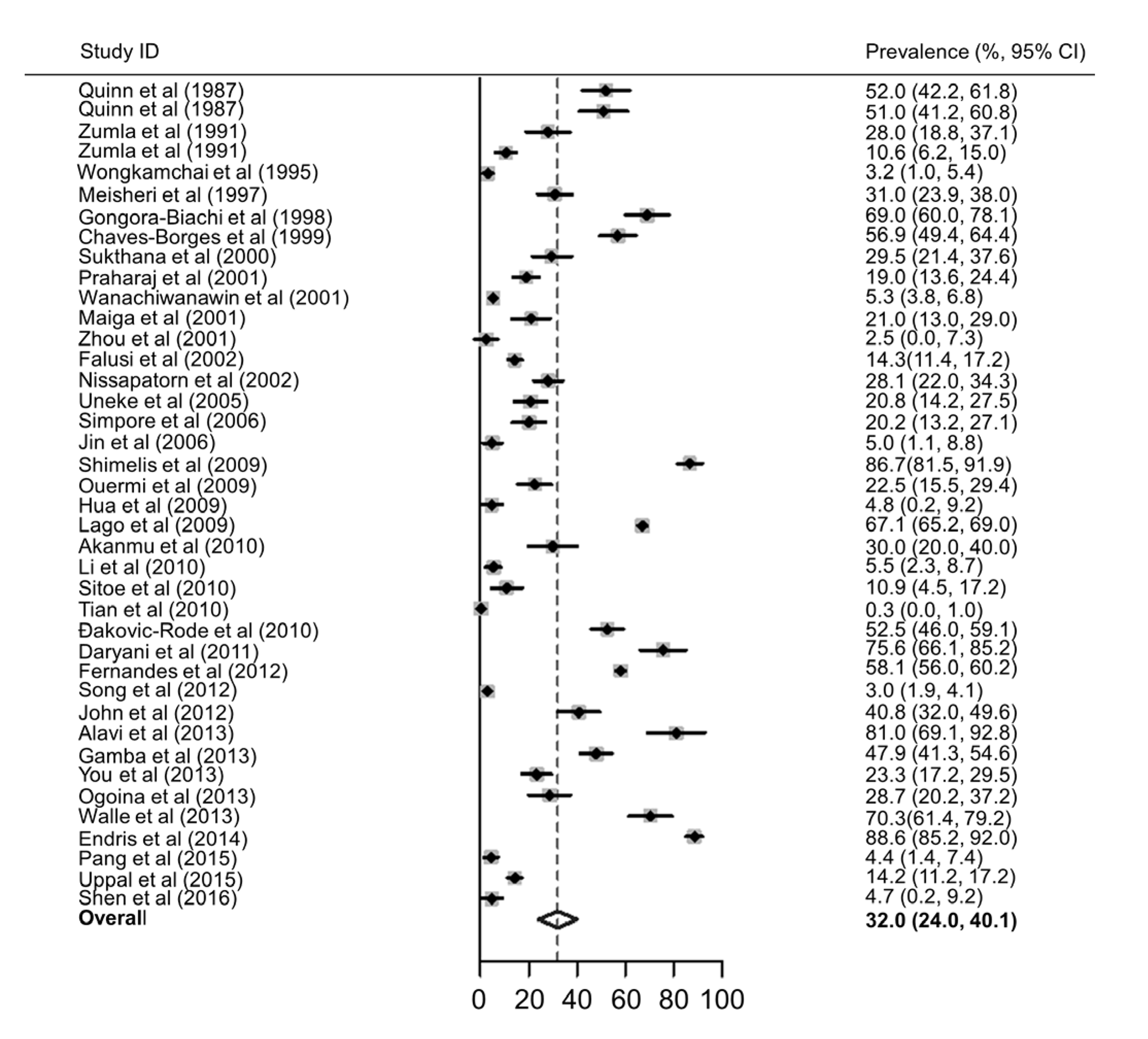

Supplement: Supplementary Figure 2 — Forest plot of estimated pooled prevalence (IgG) of T. gondii infection in HIV/AIDS-negative population. [file Image2.TIF]

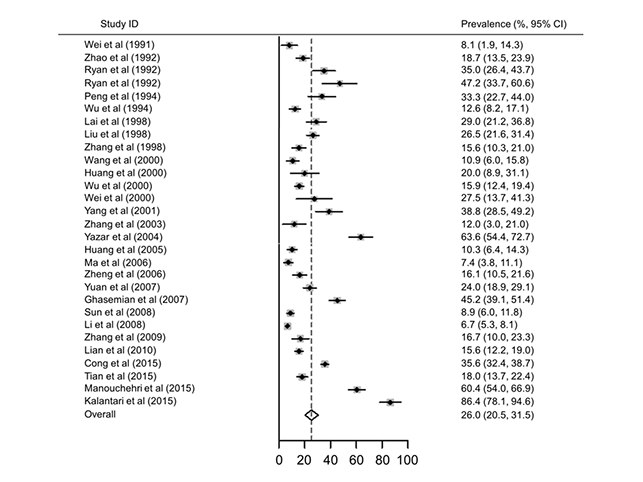

Supplement: Supplementary Figure 3 — Forest plot of estimated pooled prevalence (IgG) of T. gondii infection in cancer patients. [file Image3.tif]

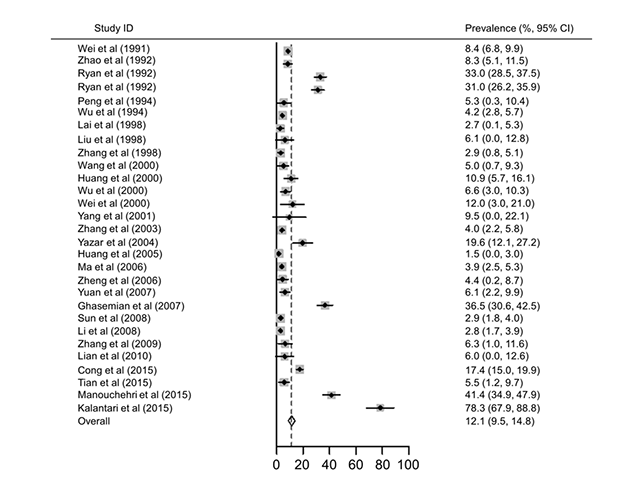

Supplement: Supplementary Figure 4 — Forest plot of estimated pooled prevalence (IgG) of T. gondii infection in cancer-negative patients. [file Image4.tif]

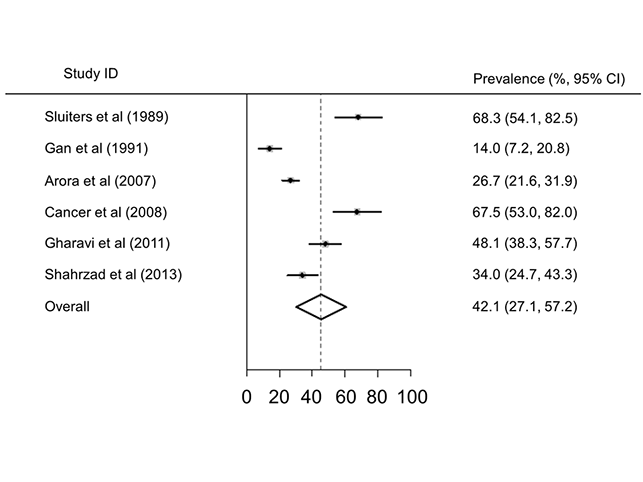

Supplement: Supplementary Figure 5 — Forest plot of estimated pooled prevalence (IgG) of T. gondii infection in transplant patients. [file Image5.tif]

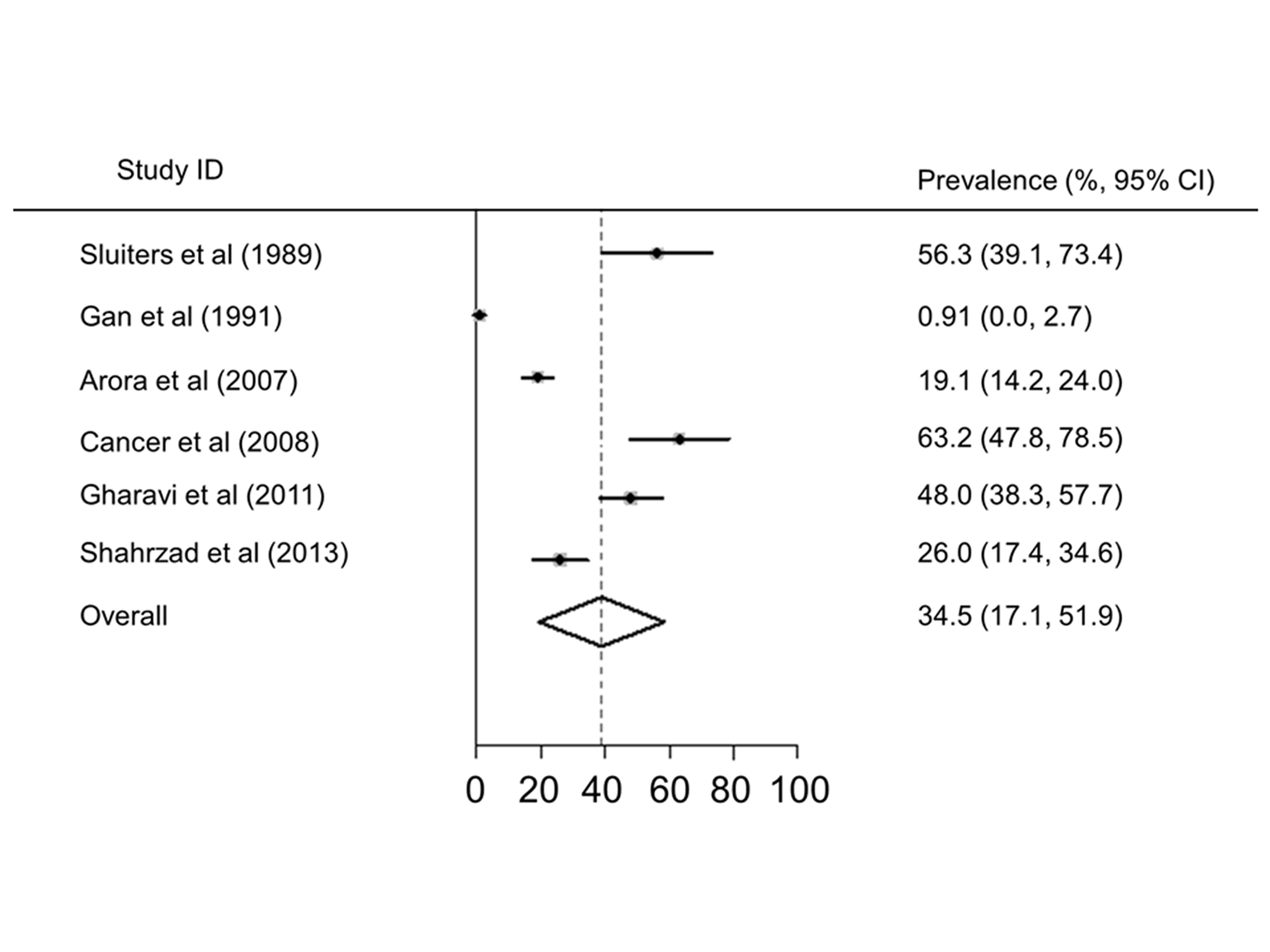

Supplement: Supplementary Figure 6 — Forest plot of estimated pooled prevalence (IgG) of T. gondii infection in non-transplant population. [file Image6.tif]

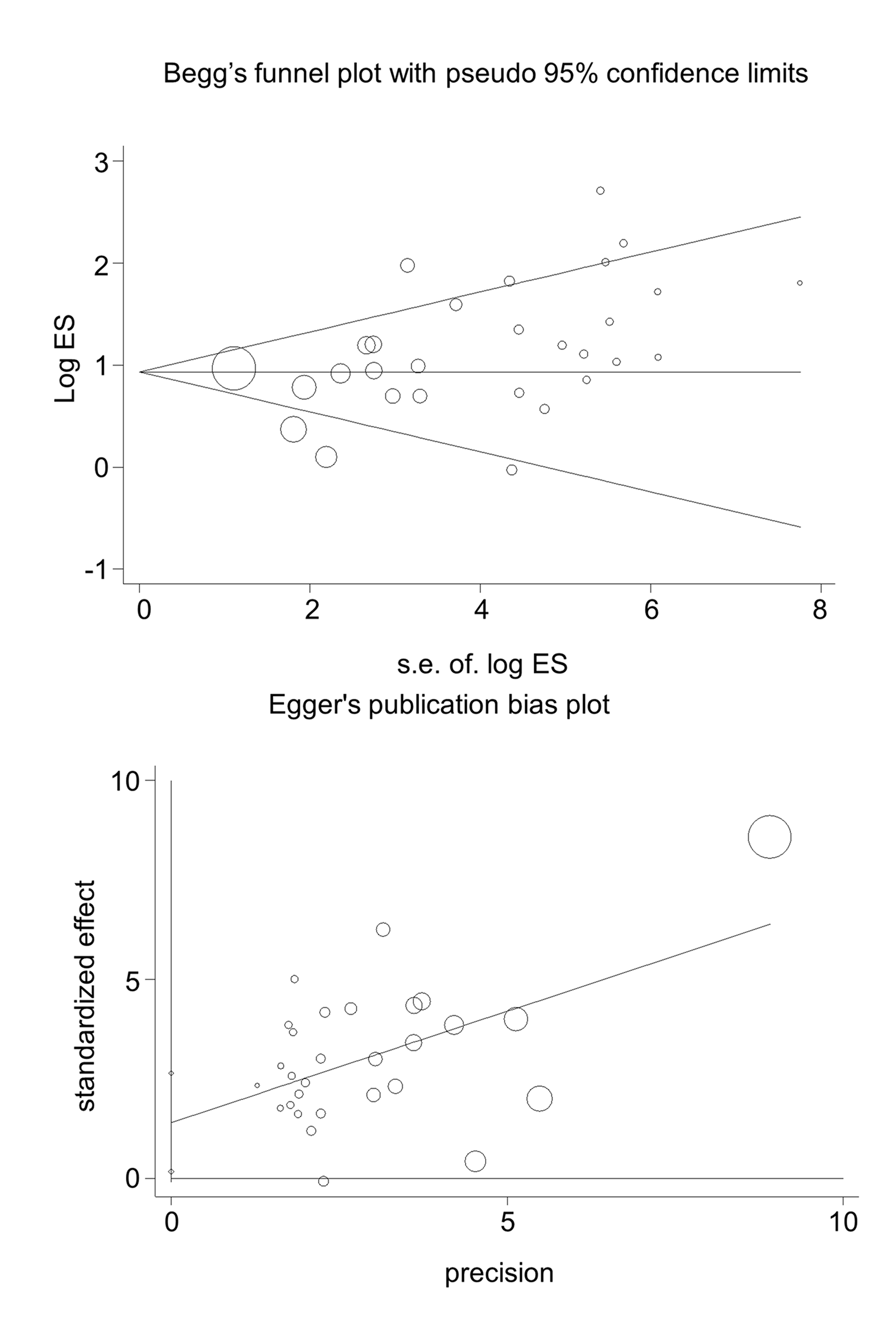

Supplement: Supplementary Figure 7 — Funnel plots to assess publication bias in the meta-analysis. (A) Funnel plot for experimental studies. (B) Funnel plot for observational studies. [file Image7.tif]

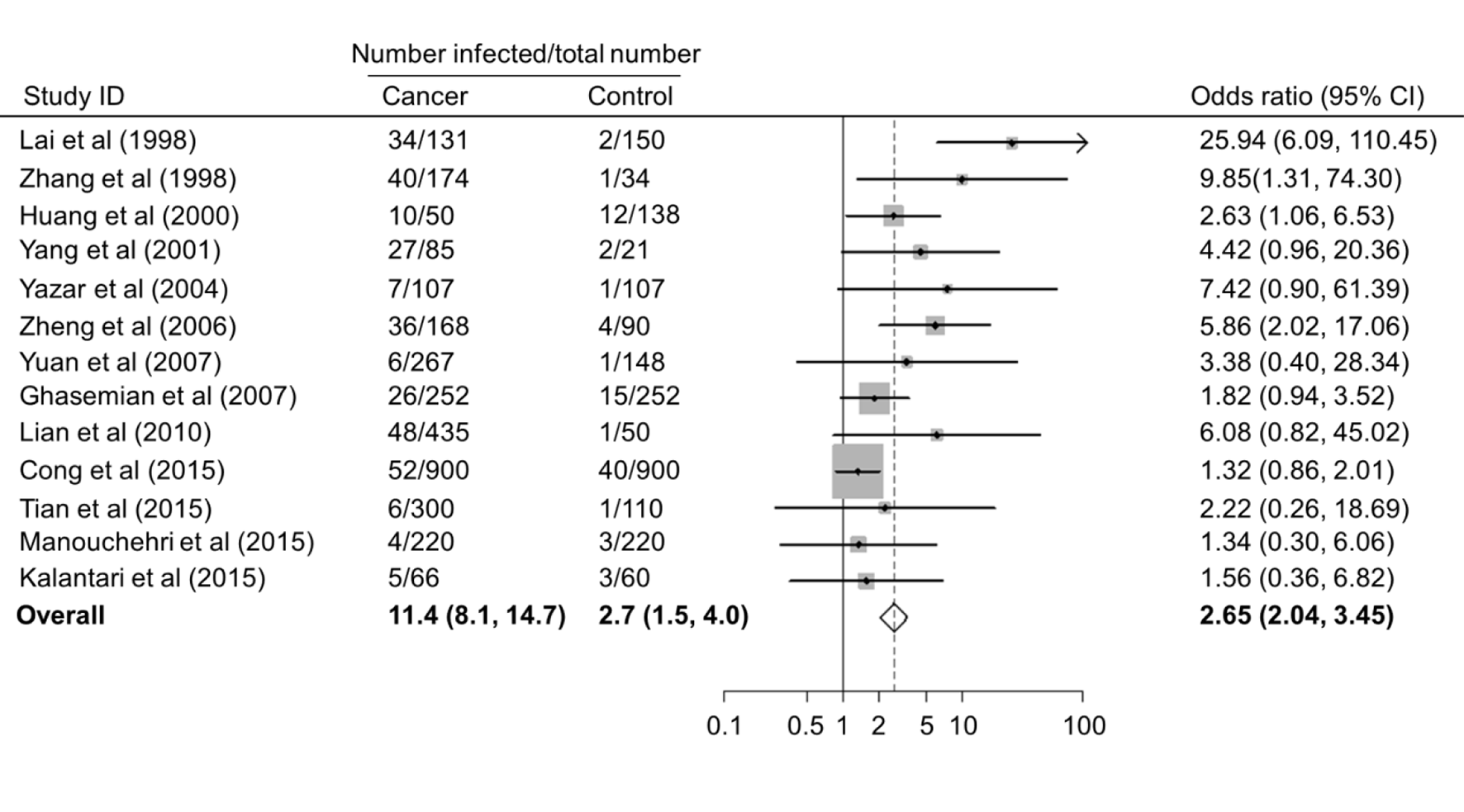

Supplement: Supplementary Figure 8 — Meta-analysis of the association of cancer patients and T. gondii infection (IgM). [file Image8.TIF]

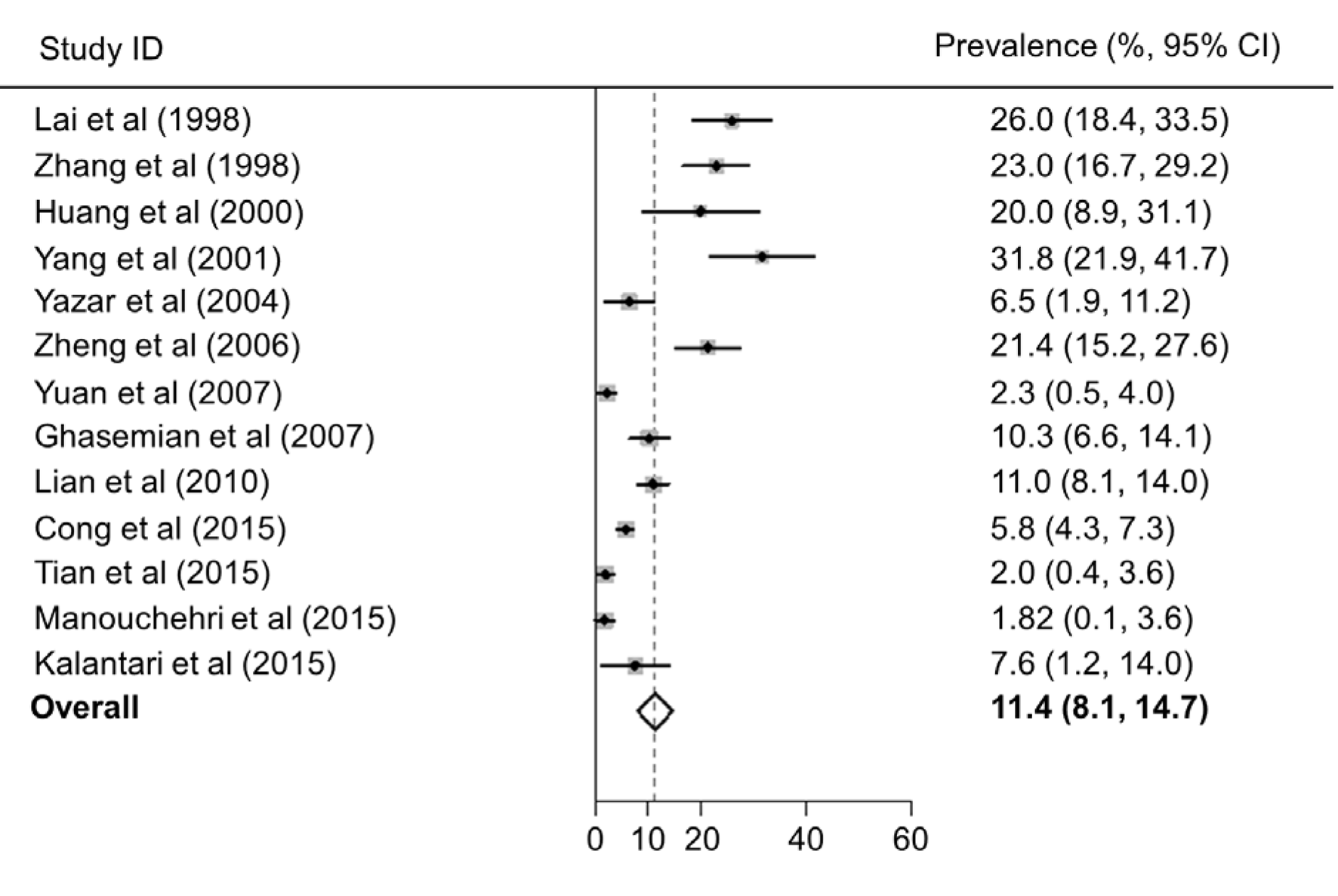

Supplement: Supplementary Figure 9 — Forest plot of estimated pooled prevalence (IgM) of T. gondii infection in cancer patients. [file Image9.TIF]

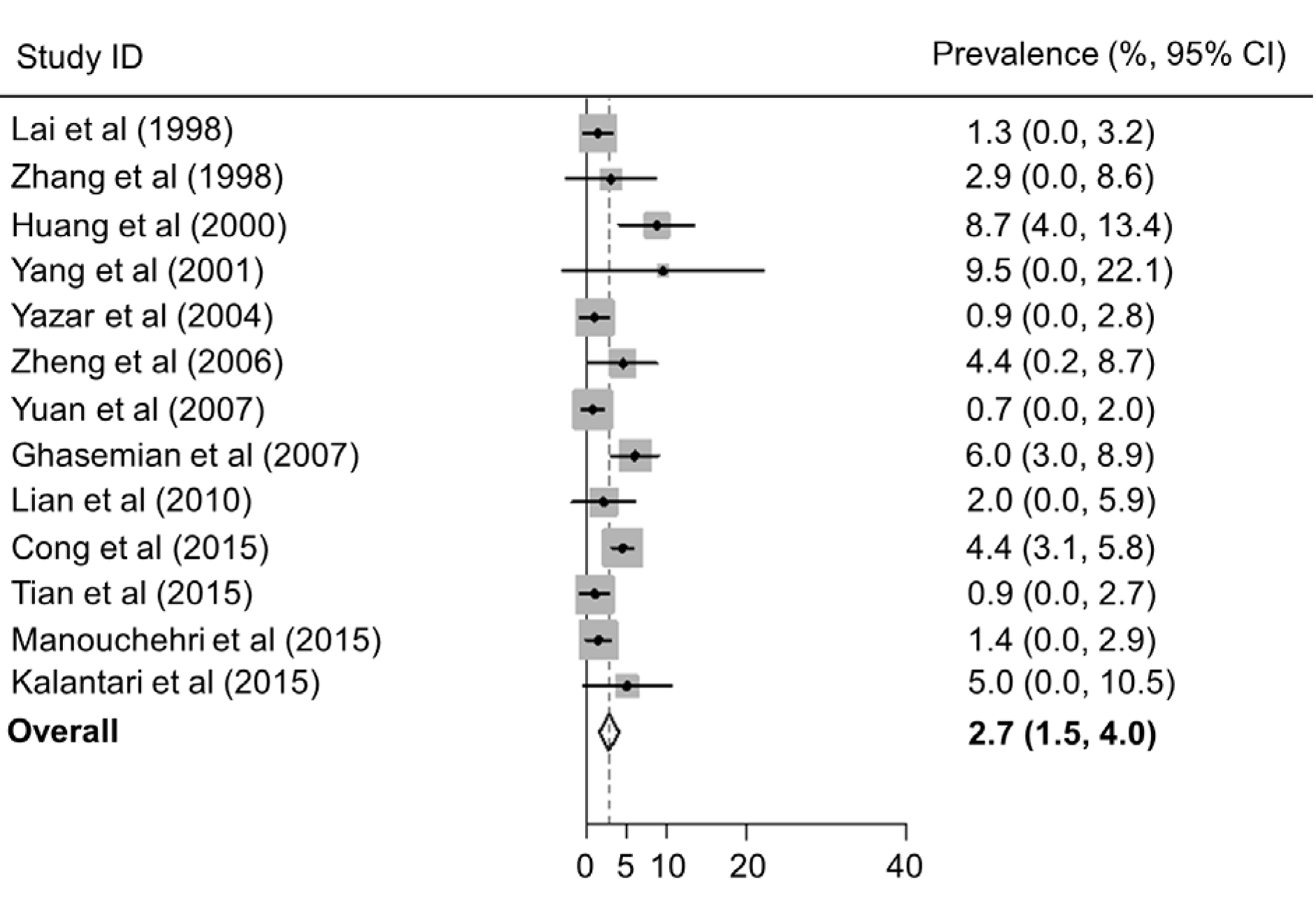

Supplement: Supplementary Figure 10 — Forest plot of estimated pooled prevalence (IgM) of T. gondii infection in cancer-negative population. [file Image10.TIF]
