# Supplementary material for: Toxoplasma gondii Infection in Immunocompromised Patients: A Systematic Review and Meta-Analysis
Source: Front Microbiol. 2017 Mar 9;8:389. doi: 10.3389/fmicb.2017.00389 (PMC5343064; doi:10.3389/fmicb.2017.00389)
Supplement: Supplementary Table 1 — Results of the subgroup analyses examining the association of immunosuppressed individuals with T. gondii infection*. [file Table1.docx]

**Supplementary table 1.** Results of the subgroup analyses examining the association of immunosuppressed individuals with *T. gondii* infection*

| **Group** | **Category** | **Subgroup** | **No. studies (datasets)** | **OR** | ***I^2^* (%)** | ***P*** | **Publication Bias Test** | |
| --- | --- | --- | --- | --- | --- | --- | --- | --- |
|  |  |  |  |  |  |  | *P* (Begg test) | *P* (Egger test) |
| **HIV/AIDS** | – | – | 40 | 1.92 (1.44–2.55) | 90.3 | 0.000 | 0.062 | 0.789 |
|  | Continent | Africa | 14 | 1.65 (1.28–2.13) | 58.6 | 0.003 | – | – |
|  |  | Asia | 19 | 2.77 (1.58–4.87) | 93.5 | 0.000 | – | – |
|  |  | North America | 3 | 0.95 (0.45–2.01) | 85.3 | 0.001 | – | – |
|  |  | Latin America | 2 | 1.19 (0.90–1.56) | 0.0 | 0.596 | – | – |
|  |  | Europe | 1 | 0.97 (0.65–1.46) | – | – | – | – |
|  |  | Oceania | 1 | 2.1 (1.34–3.43) | – | – | – | – |
|  | Income level | Low | 10 | 1.63 (1.29–2.07) | 32.0 | 0.152 |  |  |
|  |  | Middle | 27 | 2.13 (1.40–3.22) | 92.8 | 0.000 |  |  |
|  |  | High | 3 | 1.20 (0.96–1.50) | 3.6 | 0.354 |  |  |
|  | Year | 1987–1999 | 8 | 1.87 (0.86–4.06) | 91.1 | 0.000 | – | – |
|  |  | 2000–2009 | 14 | 2.11 (1.12–3.96) | 95.2 | 0.000 | – | – |
|  |  | 2010–2016 | 18 | 1.80 (1.41–2.30) | 66.9 | 0.000 | – | – |
|  | Patients | HIV / AIDS | 34 | 2.10 (1.52–2.89) | 91.3 | 0.000 | – | – |
|  |  | AIDS | 5 | 1.20 (0.71–2.01) | 66.5 | 0.018 | – | – |
|  |  | HIV | 1 | 1.24 (0.66–2.33) | – | – | – | – |
|  | Sample size | <400 | 29 | 1.81 (1.35–2.44) | 82.8 | 0.000 | – | – |
|  |  | ≥400, <1000 | 5 | 1.24 (0.66–2.33) | 49.7 | 0.093 | – | – |
|  |  | 1000≥ | 6 | 2.17 (0.87–5.43) | 97.7 | 0.000 | – | – |
|  | Methods | ELFA | 4 | 1.36 (1.02–1.83) | 23.5 | 0.270 | – | – |
|  |  | DT& LAT | 2 | 0.75 (0.22–2.60) | 83.8 | 0.013 | – | – |
|  |  | ELISA | 29 | 2.34 (1.65–3.34) | 90.8 | 0.000 | – | – |
|  |  | DT | 1 | 1.25 (0.95–1.65) | – | – | – | – |
|  |  | LAT | 1 | 0.98 (0.36–2.63) | – | – | – | – |
|  |  | MEIA | 2 | 2.06 (0.06–77.15) | 91.1 | 0.001 | – | – |
|  | Design | C–C | 26 | 1.94 (1.13–3.32) | 92.5 | 0.000 | – | – |
|  |  | C–S | 14 | 1.85 (1.36–2.53) | 77.6 | 0.000 | – | – |
|  | Population | General population | 29 | 1.66 (1.30–2.11) | 78.3 | 0.000 | – | – |
|  |  | Homosexual men | 1 | 1.66 (0.86–3.20) | – | – | – | – |
|  |  | Pregnant Women | 7 | 2.29 (0.86–6.13) | 97.1 | 0.000 | – | – |
|  |  | drug users | 3 | 3.95 (0.83–18.80) | 89.5 | 0.000 | – | – |
| **Cancer** | – | – | 29 | 2.89(2.36-3.55) | 63.4 | 0.000 | 0.028 | 0.027 |
|  | Continent | Asia | 27 | 3.07 (2.51–3.76) | 56.4 | 0.000 | – | – |
|  |  | Oceania | 2 | 1.42 (0.80–2.54) | 61.1 | 0.109 | – | – |
|  | Income level | Middle | 27 | 3.07 (2.51–3.76) | 56.4 | 0.000 |  |  |
|  |  | High | 2 | 1.42 (0.80–2.54) | 61.1 | 0.109 |  |  |
|  | Year | 1990–1999 | 9 | 3.19 (1.85–5.50) | 81.2 | 0.000 | – | – |
|  |  | 2000–2009 | 15 | 3.04 (2.28–4.04) | 55.2 | 0.005 | – | – |
|  |  | 2010–2016 | 5 | 2.51 (2.10–3.01) | 0.0 | 0.699 | – | – |
|  | Sample size | <400 | 11 | 4.21 (2.77–6.39) | 46.7 | 0.043 | – | – |
|  |  | ≥400, <1000 | 14 | 2.57 (1.95–3.39) | 63.9 | 0.001 | – | – |
|  |  | 1000≥ | 4 | 2.43 (1.77–3.34) | 48.7 | 0.120 | – | – |
|  | Methods | IHA&ELISA | 2 | 1.67 (0.62–4.52) | 71.2 | 0.063 | – | – |
|  |  | ELISA | 21 | 2.71 (2.15–3.43) | 64.0 | 0.000 | – | – |
|  |  | IHA | 5 | 4.88 (3.31–7.19) | 4.4 | 0.382 | – | – |
|  |  | ICT | 1 | 3.48 (1.56–3.95) | – | – | – | – |
| **Transplantation** | – | – | 6 | 1.51 (1.16-1.95) | 37.3 | 0.157 | 0.452 | 0.706 |

* Subgroup analysis was not performed in transplant patients, due to no heterogeneity found.
