# Supplementary material for: Toxoplasma gondii Infection in Immunocompromised Patients: A Systematic Review and Meta-Analysis
Source: Front Microbiol. 2017 Mar 9;8:389. doi: 10.3389/fmicb.2017.00389 (PMC5343064; doi:10.3389/fmicb.2017.00389)
Supplement: Supplementary Table 2 — Characteristics of the included studies for T. gondii infection (IgM) in HIV/AIDS patients. [file Table2.DOCX]

**Supplementary Table 2.** Characteristics of the included studies for *T. gondii* infection (IgM) in HIV/AIDS patients.

| **First author**  **(published year)** | **Country** | **Number infected with *T. gondii*/total number** | | **Control population** | **Method** | **Type of organ** | **Study design** |
| --- | --- | --- | --- | --- | --- | --- | --- |
|  |  | **Cancer** | **Control group** |  |  |  |  |
| Wongkamchai et al (1995) | Thailand | 0/40 | 0/248 | Normal population | ELISA | HIV/AIDS | case control |
| Zhou et al (2001) | China | 4/50 | 0/40 | Normal population | MEIA | HIV/AIDS | case control |
| Praharaj et al (2001) | India | 0/80 | 0/200 | Normal population | ELISA | HIV | case control |
| Simpore et al(2006) | Burkina Faso | 0/207 | 0/129 | Pregnant Women | ELISA | HIV/AIDS | case control |
| Ouermi et al (2009) | Burkina Faso | 5/138 | 8/138 | Pregnant Women | ELISA | HIV/AIDS | case control |
| Daković et al (2010) | Croatia | 2/166 | 2/219 | Normal population | ELISA | HIV/AIDS | case control |
| Li et al (2010) | China | 0/183 | 0/200 | Drug users | ELISA | HIV/AIDS | cross-sectional study |
| Tian et al (2010) | China | 1/302 | 0/302 | Normal population | ELISA | HIV/AIDS | case control |
| Dimie et al (2013) | Nigeria | 2/111 | 5/108 | Normal population | ELISA | HIV/AIDS | cross-sectional study |
| Fisseha et al (2013) | Ethiopia | 11/103 | 3/101 | Normal population | ELISA | HIV/AIDS | cross-sectional study |
| Shen et al (2016) | China | 3/259 | 1/85 | Normal population | ELISA | HIV/AIDS | case control |

ELISA=enzyme-linked immunosbsorbent assay. MEIA=microparticle enzyme immunoassay.

**References:**

Wongkamchai S, Rungpitaransi B, Wongbunnate S, Sittapairochana C. *Toxoplasma* infection in healthy persons and in patients with HIV or ocular disease. *Southeast Asian J Trop Med Public Health* 1995; 26: 655–58.

Zhou M, Huang X. Analysis on the co-infection of *Toxoplasma gondii* and AIDS in Xinjiang population. *Chin J Zoonoses* 2001; 17: 127

Praharaj AK, Singh SP, Chander Y, Nagendra A. Serological diagnosis of *Toxoplasma gondii* infection in various patient population in the armed forces. *Med J Armed Forces India* 2001; 57: 298–301.

Simpore J, Savadogo A, Ilboudo D, et al. Toxoplasma gondii, HCV, and HBV seroprevalence and co-infection among HIV-positive and -negative pregnant women in Burkina Faso. *J Med Virol* 2006; 78: 730–33.

Ouermi D, Simpore J, Belem AM, et al. Co-infection of Toxoplasma gondii with HBV in HIV-infected and uninfected pregnant women in Burkina Faso. *Pak J Biol Sci* 2009; 12: 1188–93.

DJakovi’c-Rode O, vZidovec-Lepej S, Vodnica Martucci M, Lasica Polanda V, Begovac J. Prevalence of antibodies against *Toxoplasma gondii* in patients infected with human immunodeficiency virus in Croatia. *Croatian J Infect* 2010; 30: 5–10 (in Croatian).

Li JR, Gong RY, Li YP, Bai Y, You F, Deng S. Research on HIV/*Toxoplasma gondii* co-infection and cytokine levels among intravenous drug users. *Parasit Immunol* 2010; **32**: 161–64.

Tian LG, Cheng GJ, Chen JX, et al. Survey on coinfection with *Toxoplasma gondii* and HIV among rural people in China. *Chin J Schisto Control* 2010; 22: 368–70 (in Chinese).

Ogoina D, Onyemelukwe GC, Musa BO, Obiako RO. Seroprevalence of IgM and IgG antibodies to *Toxoplasma* infection in healthy and HIV-positive adults from Northern Nigeria. *J Infect Dev Countr* 2013; **7**: 398–403.

Walle F, Kebede N, Tsegaye A, Kassa T. Seroprevalence and risk factors for toxoplasmosis in HIV infected and non-infected individuals in Bahir Dar, Northwest Ethiopia. *Parasit Vectors* 2013; 6: 15.

Shen G, Wang X, Sun H, Gao Y. Seroprevalence of *Toxoplasma gondii* infection among HIV/AIDS patients in Eastern China. *Korean J Parasitol* 2016; 54: 93–96.
