# Supplementary material for: Toxoplasma gondii Infection in Immunocompromised Patients: A Systematic Review and Meta-Analysis
Source: Front Microbiol. 2017 Mar 9;8:389. doi: 10.3389/fmicb.2017.00389 (PMC5343064; doi:10.3389/fmicb.2017.00389)
Supplement: Supplementary Table 3 — Characteristics of the included studies for T. gondii infection (IgM) in cancer patients. [file Table3.DOCX]

**Supplementary Table 3.** Characteristics of the included **studies** for *T. gondii* infection (IgM) in cancer patients.

| **First author**  **(published year)** | **Country** | **Number infected with *T. gondii*/total number** | | **Control population** | **Method** | **Type of Cancer** | **Study design** |
| --- | --- | --- | --- | --- | --- | --- | --- |
|  |  | **Cancer** | **Control group** |  |  |  |  |
| Lai et al (1998) | China | 34/131 | 2/150 | Normal population | ELISA | Mixed | Case-control |
| Zhang et al (1998) | China | 40/174 | 1/34 | Normal population | ELISA | Mixed | Case-control |
| Huang et al (2000) | China | 10/50 | 12/138 | Other diseases of women | ELISA | Cervical Cancer | Case-control |
| Yang et al (2001) | China | 27/85 | 2/22 | Normal population | ELISA | Mixed | Case-control |
| Yazar et al (2004) | Turkey | 7/107 | 1/107 | Normal population | ELISA | Mixed | Case-control |
| Zheng et al (2006) | China | 36/168 | 4/90 | Normal population | ELISA | Lung Cancer | Case-control |
| Yuan et al (2007) | China | 6/267 | 1/148 | Normal population | ELISA | Mixed | Case-control |
| Ghasemian et al (2007) | Iran | 26/252 | 15/252 | Normal population | ELISA | Mixed | Cross-sectional |
| Lian et al (2010) | China | 48/435 | 1/50 | Normal population | ELISA | Mixed | Case-control |
| Cong et al (2015) | China | 52/900 | 40/900 | Normal population | ELISA | Mixed | Case-control |
| Tian et al (2015) | China | 6/300 | 1/110 | Normal population | ELISA | Leukemia and Lymphoma | Case-control |
| Manouchehri et al (2015) | Iran | 4/220 | 3/220 | Normal population | ELISA | Mixed | Case-control |
| Kalantari et al (2015) | Iran | 5/66 | 3/60 | healthy women | ELISA | Breast cancer | Cross-sectional |

ELISA=enzyme-linked immunosbsorbent assay.

**References:**

Lai XQ, Zhu JY, Wei QD. *Toxoplasma* antibody in patients with malignant tumors in Yuexi area. *Guangdong Med J* 1998; 19: 281–82 (in Chinese).

Zhang WZ, Zhao JL, Qu M. Investigation of cancer patients serum antibodies to *Toxoplasma gondii*. *Chin J Parasit Dis Control* 1998; 11: 122 (in Chinese).

Huang H, Yan FH, Li CJ, Zhao MY, Tang JW, Li XR. Detection of Toxoplasma infection in women with gynaecologic neoplasms using ELISA. *Chin J Parasitol Parasit Dis* 2000; 18: 165-66 (in Chinese)..

Yang ZJ, Wang CB, Hou XF. Analysis of *Toxoplasma gondii* infection results in Malignant cancer. *Henan J Ocol* 2001; 14: 135–36 (in Chinese).

Yazar S, Yaman O, Eser B, Altuntacs F, Kurnaz F, Sahin I. Investigation of anti-*Toxoplasma gondii* antibodies in patients with neoplasia. *J Med Microbiol* 2004; 53: 1183–86.

Zheng SL, Yang QS, Ma XH. The relationship between non small cell lung cancer and *Toxoplasma gondii* infection. *Chin J Pathophysiol* 2006; 22: 1031–32 (in Chinese).

Yuan ZG, Gao SY, Liu Q, Xia XZ, Liu XF, Liu B, et al. *Toxoplasma gondii* antibodies in cancer patients. *Cancer Lett* 2007; 254: 71-74.

Ghasemian M, Maraghi SH, Saki J, Pedram M. Determination of antibodies (IgG, IgM) against *Toxoplasma gondii* in patients with cancer. *Iran J Parasitol* 2007; 2: 1-6.

Lian XW, Li KS, Du HF, Yuan M, Ye WH, Zhou YQ. Contrast analysis of antibody IgG and IgM of patients suffering different malignant tumor in Lanzhou area. *Health Vocational Educ* 2010; 28: 117–18 (in Chinese).

Cong W, Liu GH, Meng QF, et al. *Toxoplasma gondii* infection in cancer patients: prevalence, risk factors, genotypes and association with clinical diagnosis. *Cancer Lett* 2015; 359: 307–13.

Tian MY, Huang YH, Hu YF, Peng FT, Zou CY, Li YN. *Toxoplasma gondi* antibodies profile in patients with leukemia or lymphoma. *Chin J Parasitol Parasit Dis* 2015; 33: 153–55.

Manouchehri NK, Hanifepoor H, Kheiri S, Javanmardi G. Seroprevalence and risk factors of *Toxoplasma* infection in patients with malignancy in central and south central areas of Iran compared with control group using enzyme-linked immunosorbent assay (ELISA). *Trop Med Int Health* 2015; 20: 211-12.

Kalantari N, Ghaffari S, Bayani M, et al. Preliminary study on association between toxoplasmosis and breast cancer in Iran. *Asian Pac J Trop Biomed* 2015; 5: 44–47.
