# Supplementary material for: Toxoplasma gondii Infection in Immunocompromised Patients: A Systematic Review and Meta-Analysis
Source: Front Microbiol. 2017 Mar 9;8:389. doi: 10.3389/fmicb.2017.00389 (PMC5343064; doi:10.3389/fmicb.2017.00389)
Supplement: Supplementary Table 4 — Characteristics of the included studies for T. gondii infection (IgM) in transplant patients. [file Table4.DOCX]

**Supplementary Table 4.** Characteristics of the included studies for *T. gondii* infection (IgM) in transplant patients.

| Reference | Country | Number infected with *T. gondii*/total number | | Control population | Method | Transplanted organ | Study design |
| --- | --- | --- | --- | --- | --- | --- | --- |
|  |  | Cancer | Control group |  |  |  |  |
| Gharavi et al (2011) | Iran | 3/102 | 0/102 | Self-control | ELFA | Kidney | Cohort study |
| Gharavi et al (2011) | Iran | 2/102 | 0/102 | Self-control | ELISA | Kidney | Cohort study |
| Gharavi et al (2011) | Iran | 2/102 | 0/102 | Self-control | ISAGA | Kidney | Cohort study |
| Shahrzad et al (2013) | Iran | 18/100 | 4/100 | Healthy subjects | ELISA | Kidney | Cross-sectional study |

ELISA=enzyme-linked immunosbsorbent assay. ISAGA=immunosorbent agglutination assay.

**References:**

Gharavi MJ, Jalali S, Khademvatan S, Heydari S. Detection of IgM and IgG anti-*Toxoplasma* antibodies in renal transplant recipients using ELFA, ELISA and ISAGA methods: comparison of pre- and post-transplantation status. *Ann Trop Med Parasitol* 2011; 105: 367–71.

Shahrzad S, Khademvatan S, Saki J, Shahbazian H. Detection of toxoplasmosis in renal transplant recipients by ELISA and PCR methods in Ahvaz, South-West of Iran. *Jundishapur J Microbiol* 2013; 6: 1–5.
